# Nursing Perspective of the Humanized Care of the Neonate and Family: A Systematic Review

**DOI:** 10.3390/children8010035

**Published:** 2021-01-09

**Authors:** Sagrario Gómez-Cantarino, Inmaculada García-Valdivieso, Mercedes Dios-Aguado, Benito Yáñez-Araque, Brigida Molina Gallego, Eva Moncunill-Martínez

**Affiliations:** 1 Department of Nursing, Campus Toledo, Physical and Occupational Therapy University of Castilla-La Mancha, 45071 Toledo, Spain; Sagrario.Gomez@uclm.es (S.G.-C.); Brigida.Molina@uclm.es (B.M.G.); 2 Mostoles University Hospital (HMOS), Madrid Health Service (SERMAS), 28935 Mostoles, Spain; 3 Yepes Health Center, Castilla-La Mancha Health Service (SESCAM), 45313 Toledo, Spain; mded@sescam.jccm.es; 4 Department of Physical Activity and Sports Sciences, University of Castilla-La Mancha, Campus Toledo, 45071 Toledo, Spain; benito.yanez@uclm.es; 5 Toledo Hospital Complex (CHT), Neonatal and Pediatric Oncology Unit, Castilla-La Mancha Health Service (SESCAM), Theoretical Collaborator University of Castilla-La Mancha, Campus Toledo, 45071 Toledo, Spain; memoncunill@sescam.jccm.es

**Keywords:** infant newborn, pediatrics, neonatal nurses, psychosocial, critical illness, family, empowerment, nursing education, nurse training

## Abstract

This systematic review aims to determine the extent to which published research articles show the perspective of health professionals in neonatal intensive care units (NICU), as facilitators of family empowerment. Studies conducted between 2013 and 2020 were retrieved from five databases (PubMed, Cochrane, CINHAL, Scopus, and Google Scholar). The search was carried out from January to October 2020. A total of 40 articles were used, of which 13 studies (quantitative and qualitative) were included in this systematic review. Its methodological quality was assessed using the mixed methods assessment tool (MMAT). In these, the opinions and perspectives of professionals on the permanence and participation of parents were valued. In addition, the training, experiences, and educational needs of nursing within the NICU were determined. The crucial role of health professionals in the humanization of care and its effect on the neonate-family binomial was estimated. However, conceptual changes are needed within the neonatal intensive care units. To implement humanization in daily care, family participation should be encouraged in them. For this, it is necessary to modify hospital health policies to allow changes in the infrastructure that facilitate open doors 24 h a day in special services.

## 1. Introduction

The role of nursing in the care of newborns (NB) in neonatal intensive care units (NICUs) has evolved over time. This environment has a negative impact on the growth of newborns. Therefore, it is of vital importance to attenuate the stimuli, in order to favour adequate neurological development in the newborn. The newborn individualized developmental care and assessment program (NIDCAP method) aims to individualize care, observing and assessing in a comprehensive way the developmental state, and the ability to cope with the stress of the NB before, during, and after each procedure.

Currently, we are reaching a more humanized assistance and integrating the family as a fundamental part in the care of the newborn and, in turn, including them as main caregivers from birth [1,2,3]. Previously, the administration of inpatient care involved the separation of the newborn and the family. 

Even the scarce presence of parents during the estimated time of visits was perceived by health professionals as a possible risk factor for the health of the sick newborn. A matter that left parents outside the basic and technical care provided in the NICUs to their children [4,5] was that the family was considered as a stressor and not as a receiving and giving part of care [6]. Currently, the child and his/her family are perceived as an indivisible unit, recipient of care since the sick child belongs to a family with its own rules and norms [7].

For the multidisciplinary team, and in particular, for the nursing professionals, integrating the family as a fundamental part of care within the NICUs supposes a change of perspective to involve the family as the main carers. Therefore, the role of nursing has gone from being one of the main caregivers of the newborn within the NICUs to being, at present, a collaborative staff and facilitator of the empowerment of parents [2,8]. This involves the development of new knowledge, skills, and abilities for healthcare professionals, which in the past were of little importance.

The family-centered care (FCC) model carried out in various NICUs returns the importance of the neonate-family binomial as an indivisible unit to be cared for [6,9,10]. To carry out this new model of care, it is necessary to provide the multidisciplinary team that attends these units with updated knowledge, tools, and training resources to guarantee quality care based on safety and establish a relationship of trust between healthcare personnel and the family.

Among these new skills are techniques to establish efficient communication, which enables adequate health education. This provides parents with the necessary resources to carry out their role as primary caregivers [1,4,7,8]. This situation requires specialist pediatric care nursing, to ensure quality care in healthcare. In turn, it is necessary to offer strategies for coping with the stress that working in a NICU unit may entail. Multidisciplinary workspaces are also necessary, where health personnel and families contribute their vision and feel respected within it. NICU nurses positively value training in the FCC model since it is a tool that guides them in the behavioral elements to observe and, in this way, assess and plan care related to the observed behavior [3,11].

The transition to FCC in a stressful environment such as NICUs favors and improves the involvement of parents in the care of their child. It improves communication between the family and health personnel, contributes to the reduction of stress and conflicts, and favors the empowerment of the family as a care provider. This question enables technique and humanization to be harmoniously balanced [5,6,7].

The aim of this systematic review is to make the nursing perspective visible within the NICUs, in relation to the healthcare provided to the neonate–family binomial, which is a challenge within these special units, both professionally and in terms of infrastructure. It even investigates the basic and specialized training level that nurses, both new and veteran, have for the development of their skills. The nursing aptitude to function adequately is also perceived, in a highly instrumentalized environment, but where humanized care is highly valued, becoming indispensable.

## 2. Materials and Methods

A systematic review has been carried out following the Prisma guide [12], carrying out an exhaustive search in five databases (PubMed, Cochrane, CINHAL, Scopus, and Google Scholar) for articles published from 2013 to 2020. The results of the research were synthesised using strategies that avoid bias and random error. These strategies included systematic sorting of all potentially relevant articles and the description of the methodological design. They also included the analysis and the extraction of information from the articles, as well as the presentation and interpretation of the results.

The search was conducted from January to October 2020. This was due to the difficulty of including studies that reflected the experience and training of nurses within NICUs. It was also due to the need to incorporate studies that encompassed the perspective of humanization of care in terms of both nursing and family. 

Research, which includes qualitative and quantitative designs, has been used in this type of study. The search terms and threads that were used are reflected below (Table 1).

### 2.1. Selection Criteria

Papers retrieved during the searches were checked against the following inclusion criteria: (1) full-text original report published in a peer-reviewed journal; (2) articles that include the nursing perspective on family involvement in NICUs (Level I, II, and III); (3) studies indicating NIDCAP experiences and training needs of nursing; (4) research that includes the FCC model; and (5) articles written in English or Spanish.

### 2.2. Data Extraction

The search was conducted by four reviewers (S.G.-C., I.G.-V., M.D.-A., and B.Y.-A.). They read the titles and abstracts of all articles retrieved. When there were doubts about the inclusion of an article in the research, it was resolved by the consensus of the entire research team (S.G.-C., I.G.-V., E.M.-M., B.Y.-A., B.M.G., and M.D.-A.). Information about the author, year, country, study design, study purpose, sample characteristics, main variables, methodological quality level, results, and limitations was extracted from all studies. The results of studies that met the selection criteria were screened for retrieval.

### 2.3. Assessment of Quality and Level of Evidence

The quality of the selected studies was scored using a critical appraisal tool designed for systematic reviews that include qualitative, quantitative, and mixed studies and called the mixed-method appraisal tool (MMAT) [13]. The MMAT was developed in 2006, revised in 2011, and its latest version was published in 2018, which has been used in this article [13]. The list contained five items related to sample size, study measurement, design, presentation of results, and quality of research.

The total quality scores of the studies were calculated by adding up the scores of the five elements individually (range: 0–10). They were also used to categorize the level of evidence provided: studies were defined as high quality (HQ) if they had a total score of eight or more; a total score of five to seven was defined as medium quality (MQ); a score below five was defined as low quality (LQ).

Four reviewers (S.G.-C., I.G.-V., M.D.-A., and E.M.-M.) assessed study quality separately. In addition, a meeting was held to resolve possible disagreements between all the reviewers (Table 2).

## 3. Results

### 3.1. General Findings

Once the selected articles were evaluated, it was found that of the 13 included studies, six (46.14%) obtained a score of between 8–10 points, which indicates their high quality [15,17,20,24,25,26]. Three of them (23.07%) belonged to qualitative studies, and another three (23.07%) belonged to quantitative studies. On the other hand, five (38.46%) articles were classified as medium quality studies [14,16,18,21,23]. Two (15.38%) of these articles belonged to qualitative research, while three (23.08%) belonged to quantitative studies. Only two (15.38%) studies obtained a score indicating low quality after being analyzed [19,22]. One (7.69%) was a descriptive qualitative study and one (7.69%) a quantitative study.

**Figure 1:**
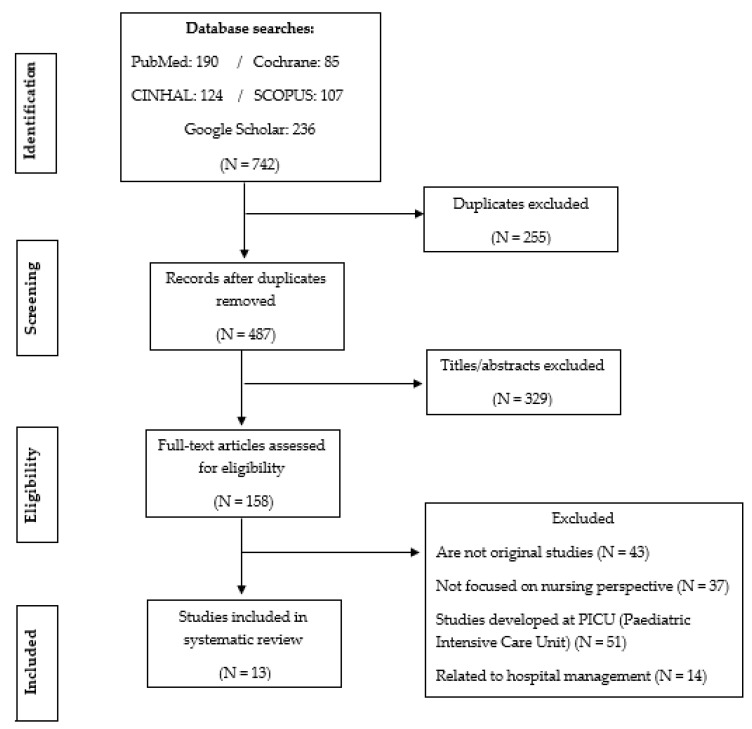
The flow of articles through the search process.

The flow of search results through the systematic review process is displayed in PRISMA. The initial search retrieved 742 articles, which were reduced to 487 by eliminating duplicates. The titles and abstracts of these 487 studies were screened, resulting in the exclusion of 329 additional studies. Of the 158 remaining, 145 were excluded because they were not original studies, did not focus on the nursing perspective and were developed in the pediatric intensive care units (PICU), and were related to hospital management. Thus, 13 studies were included in the systematic review (Figure 1).

Four studies [15,16,21,24] were conducted in hospitals with high technology (*n* = 6), five studies [14,19,22,25,26] were conducted in general hospitals (*n* = 14), and four studies [17,18,20,23] in medium-sized hospitals (*n* = 82).

Regarding the types of studies selected, we found that seven (53.84%) are quantitative studies, of which two (15.38%) were descriptive, two (15.38%) were multicenter, one (7.69%) was cross-sectional, one (7.69%) was non-experimental, and one (7.69%) was logistic regression. A total sample of quantitative studies of *n* = 2042 (92.94%) was obtained. Regarding qualitative research, six (46.16%) studies were selected, since they met the inclusion criteria, with a total number of participants of *n* = 155 (7.06%). The sample size of the studies ranged from 10 [22] to 372 nurses [18]. The samples were collected from seven different countries: one study in Ireland, three in Spain, one in Sweden, one in Turkey, three in Iran, two in Finland, and two in the USA. Table 3 shows the main characteristics of the selected studies with the participating health professionals.

### 3.2. Health Professionals’ Perspective on Parental Involvement

Once the gender variable was analyzed, the selected articles provided significant data, yielding a total sample of *n* = 2362 professionals. Of these, *n* = 2197 (93.01%) were women, while *n* = 165 (6.99%) were men. The latter professionals were related to the NICU, both because they were specialists in neonatology and because they were hospital directors [18]. Therefore, it can be affirmed that the presence within the nursing profession is mostly female because it is traditionally and culturally linked to care. Even within these units, it is valued that the female presence is significantly higher compared to the male presence [18].

On the other hand, the age variable provides relevant information since in some of the studies carried out in countries such as Ireland [14], Spain [15,16,17], Sweden, [18], Iran [20], Finland [21], and the USA [22], it was observed that the mean age of health professionals is approximately 30–40 years. This situation plays a prominent role in encouraging parents to be close to their newborn. From the selected studies, it is highlighted that Spain, Finland, and Ireland [14,15,16,17,21] have a younger nursing population, aged 30 years (*n* = 259). On the other hand, it is seen that Iran and the USA [20,22] have a mean age of 40 years (*n* = 43), while Sweden [18] has the oldest nursing group with 40–50 years (*n* = 372) of a total of *n* = 674 nurses. Research shows that the youngest professionals [16], although focusing on the family as a unit of care and even showing respect for their preferences, are more focused on technology, with their attention to the family being in the background. On the other hand, middle-aged staff [20] promote interpersonal relationships, an issue that increases family capacities in the care process. It is clear that the most organisational level dedicated to management is developed in studies where the sample is larger [18], as services are coordinated and favourable environments are provided within the unit itself. Even decision-making is coordinated among the multidisciplinary team including the family.

The youngest (38.42%) and middle-aged (6.37%) healthcare professionals who participated in the studies report that cultural differences or language barriers are diluted through nurse–family participation in the NICU. This is due to the fact that the parents observe the progress of the newborn through their participation in the care. Support groups for parents are also promoted, where similar circumstances are addressed both in pathologies and essential care for the upbringing of their children once they are discharged from hospital [15,18,21].

In this sense, the information provided by quantitative studies [18,19] is relevant, since they discover that the participation of parents within the NICU is necessary for the development of newborn rearing skills. For such participation to occur, NICU nurses must be up-to-date in care focused on the neonate–family binomial [19]. To further this finding, a study conducted in Finland shows that well-trained nurses (*n* = 22 NICU nurses N-III) facilitate the establishment of a family-centered culture of care [21]. Thus, from the point of view of these professionals, the participation of parents within the NICU manages to raise the quality of care, allows greater confidence in their professional role, and achieves satisfaction with the work performed [20].

The qualitative study carried out by Toivonen [23] in which *n* = 51 professionals (*n* = 32 nurses and *n* = 19 medical managers) participated, shows that, with the participation of parents in the NICU, they are the ones who manage to perform basic care that facilitates the comfort of the newborn. This fact allows them to perceive the comfort of their newborn and consequently reduce their level of stress, which is inherent in hospitalization [20]. In this sense, it should be noted that parental participation modifies the nursing role because, through the involvement of parents, an atmosphere of complicity with the nursing staff is generated in the NICU. In this way, the nursing role goes from being an active caregiver to a support facilitator for the newborn’s parents, an issue visible in the study carried out in Finland [21].

The information provided by a qualitative study conducted in Iran in 2020 is worth dwelling on [26]. This research affects the need for education and training of nursing staff (*n* = 25) to carry out FCC. They even insist on training to carry out care with the mother present, using the kangaroo method. Currently, health personnel (*n* = 40 nurses and neonatologists) must provide instruction, training, and even teach parents of different cultures, beliefs, and socio-cultural levels, while carrying out their care work.

However, it is interesting to highlight another qualitative study also conducted in Iran in 2019 [25], (*n* = 120 nurses) that warns that parental involvement is not a new concept in the field of newborn care, although this practice is being implemented ideally in many countries [18,19]. Nevertheless, it is true that the study carried out in Iran [25] shows that changes in the health policies of the center will be necessary if a hospital does not have adequate resources that allow the development of a culture of care centered on the family. This can translate into increased funding for increased staffing, NICU renovation, ongoing staff training, and even parent accommodation.

### 3.3. NICU Nursing Training, Needs, and Experience

Of the 13 studies selected, nursing experience or specialization within the NICU is reflected in nine (69.23%) of the studies reviewed, with a total sample of *n* = 1.346 nurses. Research has revealed that nurses must be trained to educate parents on the most appropriate ways to care for their newborns. Care improves when it is offered by nursing with more years of experience and better training. Thus, the research reviewed indicates that *n* = 342 nurses have a range of work experience of 0–5 years. While *n* = 812 nurses have an experience of 5–15 years, and *n* = 192 nurses are providing their services in the NICU for a period longer than 15 years [15,16,17,20].

Therefore, the nursing group that has an average of 5–15 years worked (14.26%), guarantees that the time factor is comparable with better training, which results in a higher quality of patient care and interventions in the neonate without forgetting that new nurses and those with less experience within the NICU also have training in FCC [15,16]. This situation highlights the need to update knowledge with an FCC approach, which should be mandatory within special services, involving the entire multidisciplinary team including psychologists [17,20].

In this sense, an investigation carried out in Sweden in 2017 with *n* = 443 professionals clarified that, for the medical profession, this participation gained importance as the FCC culture was introduced in the NICU [18]. Therefore, according to this study, physicians (*n* = 71) should also undergo FCC training periods in addition to *n* = 372 nurses. On the other hand, an investigation reveals how the most experienced nursing personnel provide care to the newborn from the first moment of their admission to the NICU, focusing their attention on the neonate–family binomial, while the newer personnel focus their immediate attention on the newborn despite having the necessary training in FCC [19].

### 3.4. Humanization of Care in NICU: Promotion of the Nurse-Family Relationship

Among the 13 documents selected to carry out this systematic review, there are outstanding investigations focused on the humanization of care in the NICU. Specifically in the USA, the studies carried out by Coasts [22] and Gilstrap [24] refer to the need for *n* = 24 nurses to establish a positive relationship with parents, which makes it possible to humanize the personnel–family relationship within the NICUs.

Through this mode of relationship, an atmosphere of cordiality is generated between the nursing staff and the parents. Therefore, when delving into this sense, one of the investigations [24] clarifies that, to establish this relationship with the parents, it is essential to avoid the rotation of the personnel assigned to care for the newborn at least for a period of six months. Thus, of the *n* = 14 nurses in the study, *n* = 10 (71.42%) had day shift, *n* = 2 (14.28%) night shift, and *n* = 2 (14.28%) performed both shifts. It is appreciated that unnecessary rotations can hinder the beginning of trust between the nursing staff and the family. This being essential to start and maintain a positive nurse–family relationship. Even the change of shifts (morning, afternoon, and night) can deteriorate a previously built relationship between the nursing staff and the family.

In a qualitative study of *n* = 22 nurses, it is valued that humanized care is enhanced when parents are allowed to spend more time in the NICUs [21]. This issue favors greater participation in basic care and a close, more effective relationship with the nursing staff. This relationship between nursing and family allows us to express the suffering and concern accumulated by each of the members of the family unit [18,21]. In a study carried out in Sweden [18] with a sample of *n* = 443 health professionals, of which *n* = 372 (83.97%) were nurses, it can even be seen that the greater the experience of the health professionals within these units, the greater the relationship with the family is promoted. On the other hand, the study carried out in Finland [21] indicates that there is a greater understanding between the more mature nurses and the family, achieving a pleasant and trusting atmosphere within the NICUs. This promotes smooth communication with parents, even when their children are admitted to intensive care [21]. This situation leads to positive feedback between staff and family [18,21].

One of the research conducted in the USA in 2018 with *n* = 10 nurses states that the FFC model promotes open and inclusive communication between nursing and the family. The family gradually loses its fear of the newborn’s fragility [22]. This allows them to receive real-time information about their child’s health status.

This mitigates their hopelessness and manages to humanize care within the unit [22]. It is also appropriate to clarify that nursing demands to be an active member of health policies that promote the permanence of the family within the NICUs. Through the FCC, parents are encouraged to participate in the care of their newborn. Even one of the qualitative studies carried out in Finland in 2019 with a sample of *n* = 32 nurses involved the family in carrying out basic tasks of caring for the newborn since, upon discharge from the hospital, the parents will carry out such care at home [23].

However, not all nursing personnel (*n* = 42 nurses) are motivated to carry out their work activity in the presence of the family within the NICU. On occasions, parents do not stop asking questions, which prevents the performance of techniques between the staff and even when it is vital to perform emergency care in a critical situation [22]. The influence of the environment of these units must be taken into account. Even the stress that can be generated among nurses and family members makes the communication strategies used by these professionals very important, without forgetting that the number of newborns to be cared for, their weeks of gestation, and the number of nurses within the unit and their levels of experience will also have an influence [22,23].

Therefore, in relation to the humanization of care in the NICUs, some of the investigations warn that it is necessary to adapt to private spaces where parents can be alone with their children [22,23]. This means adapting the units with spaces where information can be transferred between professionals, in turn, equipped with technology that allows continuous supervision and monitoring of the newborn as a safety tool. Which facilitates the family privacy necessary to establish the parental role [23].

It is worth dwelling on the information provided by a study carried out in Ireland in 2013, where the opinion of *n* = 250 nurses from seven hospitals was collected. They considered that financial support to families is essential to implement the humanization of newborn care. Because of the expenses that newborn care represents for the parents, their travel to the hospital, personal hygiene, and maintenance during the admission of their child can have an economic impact for families that is difficult to assume [14].

The *n* = 53 nurses, belonging to four NICUs (with care experience between 10–11 years) who participated in the study carried out in Turkey in 2017, reported that to implement the FCC model, the first point where the institution must intervene is with personnel, who must be trained and motivated to develop their professional practice within these units. The workload that professionals have within the unit in relation to the care of the newborn will not be an obstacle, but it will be a driving factor to change the professional role for the different levels that make up the units. A greater approach is even valued in nurses (with a mean age of 32 years) who have their own family and descendants [19].

## 4. Discussion

The objective of this systematic review was to examine the perspective of healthcare personnel in NICUs as facilitators of family empowerment. A total of 13 studies were selected that met the inclusion criteria, which were conducted from 2013 to 2020. They were diverse in their methodologies (quantitative and qualitative). The results obtained were related to the perspective of the professionals in the administration of care, the need for more specialized training within special units such as the NICUs, and the more humanized contact between the professionals and the family.

In relation to the gender variable, the data showed that, at present, the presence of females continues to be much higher than males in the nursing profession. In fact, some studies value that the female nurse figure incorporates concepts such as participation and negotiation to enhance family empowerment [4,6]. Nursing even covers the demands of the family by including them in the care plan and detecting their needs [27].

The age variable reflected that the largest group is the one with more years of experience in complex units such as the NICU. Specifically, this group of nurses has an age between 40 and 50 years. The results support that these years of care practice play a crucial role in training parents as primary caregivers [28,29]. Curiously, it is found that the newest nurses are those with the most university training, an issue that encourages family participation, although it is true that since they do not have enough work experience, they focus more on offering care technicians to the newborn [15,16,17,20].

However, in recent decades, a training process has been favoured for university graduates, with a command of the scientific method and with a multi-professional approach, which allows greater understanding, interpretation, and solution of the problems related to their healthcare activity [23]. For its part, another study [28] considered that permanent training and systematic updating of professionals within special units leads to the improvement of job performance. This encourages professional improvement to develop through a set of organizational forms that complement and enable the study and dissemination of social, scientific, and technological advances that influence better healthcare [23].

In recent decades, there has been a movement to strengthen improvement in nursing, recognized through the World Health Organization (WHO), which gives a strategic character to its actions. This scenario allowed the emergence of postgraduate nursing programs in different countries [30]. In fact, in Spain, one can speak of the specialty in pediatric nursing through the internal resident nurse (EIR) system. In these studies, nurses received specific training related to family involvement in neonatal care [20,26].

Therefore, the process by which nursing decides to specialize closely relates to Benner’s theoretical model, in which the process followed by a healthcare professional is exposed until specialization in a specific area is reached [31]. This theory even shows how nursing goes through a series of stages until reaching the expert level (beginner, advanced beginner, competent nurse, efficient nurse, and expert nurse) [32].

In this sense, it is found that the expert nurse resolves critical situations, strives to improve care, and promotes changes in daily routines, achieving patient and family satisfaction [31,32,33]. It corresponds to the nurse involved in the formation of the family, as it is an indispensable pillar for the care of the newborn [15,16,17]. In this way, parents become protagonists of the care process, promoting much more active experiences [26,30,34]. This philosophy considers that the nursing team in the NICUs must have an integrative vision that combines scientific, technological, human, and emotional aspects, in continuous evolution aimed at excellence in care [31,32].

Currently, there are numerous demands in Spain on the part of parents regarding the extension of hours within the NICU. Even in 2013, an agreement was reached between the Ministry of Health and the Autonomous Communities (CCAA) to promote the opening of NICUs 24 h a day, although this situation in Spanish hospitals is not fully met [35]. Even though it is true that there is controversy among professionals, it is found in the reviewed studies that healthcare professionals see it as adequate to provide support to the family, but their desire not to involve them in care is also perceived [18,19,20]. However, other studies affirmed that the professionals consider parental presence beneficial and adequate, both for the neonate–family binomial and even for the nursing staff, reducing their level of stress [21,22,23]. Nevertheless, the change that is taking place towards open-door NICUs enables a transformation towards the humanization of care [36]. To provide adequate care for the newborn and the family during hospitalization, it is necessary that there be a good nurse–patient relationship, and an adequate number of them in work shifts (morning, afternoon, and night). This even enhances the continuity of care by the same professional, avoiding unnecessary shift rotations. These services guarantee the provision of comprehensive and continuous care to the critically ill neonate [24,37,38]. Therefore, nursing within the NICU must promote a new paradigm where holistic and global care meets the needs of the newborn and the family, in one of the most critical moments after birth [28,33,39,40].

Measures that can be applied in NICUs are proposed to enhance the humanization of newborn and family care are as follows:

(1) Health policies should be promoted that allow hospitals to remain open 24 h, where the family’s presence can be uninterrupted. This issue promotes their inclusion within the healthcare team.
(2) Healthcare management should promote the inclusion of healthcare professionals (nurses and doctors) with specific training in this type of unit and even promote learning courses for new professionals who join these units and become part of the multi-professional team. This situation would contribute within the NICUs to joint participation of the multidisciplinary team and the family.
(3) The humanization within the NICUs should be addressed from the beginning by the professionals themselves, encouraging parental participation and giving meaning to the experiences of families. This situation can be carried out through the management area, respecting the shifts of the nursing that is found in these units.

## 5. Conclusions

Advances in hospital care have led to a new paradigm in the way of caring, in which the parents’ involvement in care and their permanent presence during medical and nursing procedures is considered beneficial for both the family and the newborn. This situation implies the need to establish individual rooms, each one with a bed, to offer the family rest. A common room for families is also promoted, which encourages the relationship between different families. In addition, this space is usually equipped for use during meals. Thus, for these families, the hospital stay can be maintained over time due to the economic savings that these measures represent.

However, there is still no general vision to apply this new way of working where the presence of parents is formalized 24 h a day. More training would be necessary for both the healthcare personnel and the family itself. Even health policies, such as health managers, should include improvements regarding space and trained personnel in these units. In addition, the presence of an intermediate command 24 h a day would be necessary to coordinate the health personnel with a unified turnaround where the routines facilitate the care of a neonate in a critical situation.

Therefore, it is verified that the patient and his/her family are recognized as the focus of attention. That nurses and doctors, as well as other professionals of the health team, must actively participate in humanized care. In addition, the patient and her/his family must be included in decision-making, and may even discuss the daily care plan and the expected results. This issue enhances family participation in the continuity of care when they are applied by the same professionals in their corresponding shifts within the NICUs. Even these professionals know the preferences of the newborn and the family for greater involvement in care. Therefore, care centered on the family and the newborn becomes safer, more efficient, effective, and timely.

## Figures and Tables

**Table 1:** Search strategy in databases.

Database	Search Strategy	Limits	Filters
PubMed	Infant newborn OR Pediatrics AND Neonatal nurses OR Caregivers AND Critical care OR Critical illness AND Family AND Empowerment AND Psychosocial AND Nursing Education AND Nurse Training	Title Article English/Spanish	190 items filtered
Cochrane			85 items filtered
CINHAL			124 items filtered
Scopus			107 items filtered
Google Scholar			236 items filtered

**Table 2:** List of included studies with quality scores.

Author(s)	A	B	C	D	E	Total Score	Quality Level
Coyne et al. [14]	2	1	1	1	1	6	MQ
Mosqueda et al. [15]	2	2	2	2	1	9	HQ
Mosqueda et al. [16]	2	2	1	1	1	7	MQ
Mosqueda et al. [17]	2	2	2	2	1	9	HQ
Kjellsdotter et al. [18]	2	2	1	1	1	7	MQ
Kucuk et al. [19]	1	1	1	1	0	4	LQ
Baghlani et al. [20]	2	2	2	1	1	8	HQ
Axelin et al. [21]	1	1	2	2	1	7	MQ
Coasts et al. [22]	1	1	1	2	0	5	LQ
Toivonen et al. [23]	1	2	2	1	0	6	MQ
Gilstrap et al. [24]	1	2	2	2	1	8	HQ
Heidari et al. [25]	1	2	2	2	1	8	HQ
Mirlashari et al. [26]	1	2	2	2	1	8	HQ

HQ: high quality; MQ: medium quality; LQ: low quality. A: sample size (2: more than 100 participants; 1: 10 to 99; 0: fewer than 10 participants); B: study measurement (2: suitable; 1: not very suitable; 0: nothing suitable). C: design (2: suitable; 1: not very suitable; 0: nothing suitable). D: presentation of results (2: relevant; 1: not very relevant; 0: not relevant). E: quality of research (2: very good; 1: good; 0: low).

**Table 3:** Characteristics of the studies showing: perspective, training, and humanization of nursing.

**Quantitative Studies**						
**Author(s), Year, and Country**	**Study Design**	**Study Purpose**	**Sample Characteristics**	**Main Variables**	**Results**	**Limitations**
Coyne et al. (2013) Ireland	Quantitative Non-Experimental Survey	Investigate perceptions and practices of nurses about FCC and examine the influencing factors.	*n* = 250 NICU nurses *n* = 7 hospitals	- Age: 20–30 years (88) 31–40 (114) 41–50 (38) >50 (10) - Gender Female (236) Male (14) - Work experience 0–5 (68) 5–15 (106) 15–20 (36) >20 (40) FCCQ-R Questionnaire: - Family-professional collaboration - Family recognition - Emotional support - Shared information.	Work experience is related to more positive support for family involvement. Updating knowledge helps nurses to apply the FCCs, but they are not able to apply all the elements due to lack of resources, organizational barriers, hospital design.	Small sample size. FCCQ-R questionnaire still under development. Low response rate (33%). Only nurses’ experience was taken into account, not families or other professionals.
Mosqueda et al. (2013) Spain	Quantitative Multivariate Logistic Regression Analysis	Identify: requirements, professional perceived barriers (NIDCAP)	*n* = 305 professionals *n* = 164 professionals NICU N-III Madrid (H. 12 Octubre) *n* = 141 professionals NICU N-III Barcelona (H. Vall d’Hebron)	- Sociodemographic: Profession: Neonatologists (40) Nurses (169) Assistants (94) No response (2). Age: 20–35 years (139) 36–50 (119) >50 years (46) No response (1). Gender: Male (23) Female (280) No response (2). Time worked <5 years (135) 6–10 (68) >10 years (58) No response (44). - Information sources. - Necessary resources. - Perceived barriers.	Knowing the opinions of professionals makes it possible to improve conditions and facilitate work.	Carrying out the study in the middle of the implementation period can influence the perception of requirements and barriers.
Mosqueda et al. (2013) Spain	Quantitative Descriptive	Explore professional perception of NIDCAP application.	*n* = 305 professionals *n* = 164 professionals NICU N-III Madrid (H. 12 Octubre) *n* = 141 professionals NICU N-III Barcelona (H. Vall d’Hebron)	- Sociodemographic: Profession: Neonatologists (40) Nurses (169) Assistants (94) No response (2). Age: 20–35 years (139) 36–50 (119) >50 years (46) No response (1). Gender: Male (23) Female (280) No response (2). Time worked <5 years (135) 6–10 (68) >10 years (58) No response (44).	No differences in perception regarding the gender variable. Younger professionals more positive assessment of NIDCAP. Professionals H. Vall d’Hebron highest scores. Neonatologists perceive NIDCAP more positively, nurses feel it requires more time to implement. NIDCAP improves parent-professional relationship.	For nursing, it means a greater workload. Carrying out the study in the middle of the implantation process can influence the results obtained.
Mosqueda et al. (2016) Spain	Quantitative Observational Multicenter	Determine: theoretical-practical course on individualized care (NIDCAP) effect: degree of knowledge and professional satisfaction	*n* = 566 professionals *n* = 20 NICUs (N-III)	- Level of knowledge before and after the course, with a questionnaire of 30 questions. - Course satisfaction.	The course improved the level of knowledge. The participants expressed a higher level of satisfaction.	The participants knew they were being watched and evaluated. Since the questionnaires were anonymous, they did not allow evaluating the pre and post levels of each participant or professional group. Inability to evaluate aspects such as acquired skills, attitude change and impact on patients.
Kjellsdotter et al. (2017) Sweden	Quantitative Transversal	Examine age, gender and profession association regarding importance of NICU parental care participation.	*n* = 443 professionals (*n* = 372 nurses, *n* = 71 physicians) *n* = 29 NICUs.	- Age - Gender - Profession - NICU experience - Hours worked week - Parents involvement	Nursing believes that the involvement of parents is important.	Non-representative sample of NICU professionals. The validation of the questionnaire used is questioned. Personal factors affect how you respond.
Kucuk et al. (2017) Turkey	Quantitative Descriptive	Know perception nurses working in NICU on family-centered care.	*n* = 53 nurses *n* = 4 NICUs.	- Age - Education level - Marital status - Nº. children - Work experience - FCC knowledge	It is necessary to increase the number of nurses who participate in the elaboration of protocols, to increase the implementation of the FCC. Educational level, marital status and having children positively influenced nursing perception.	It has no limitations.
Baghlani et al. (2019) Iran	Quantitative multicenter	Evaluate knowledge, perception nursing (NIDCAP method)	*n* = 120 NICU nurses.	- Sociodemographic: Marriage Nº of children Level of studies Work field Work shift	Excellent knowledge and perception of nursing. Greater satisfaction and more positive attitude	The self-report method of conducting questionnaires may not express reality. Limited sample size
**Qualitative Studies**						
**Author (s), Year and Country**	**Study Design**	**Study Purpose**	**Sample Characteristics**	**Main Variables**	**Results**	**Limitations**
Axelin et al. (2014) Finland	Qualitative	Describe nursing experiences: training parents influence in the NICU.	*n* = 22 nurses (NICU N-III)	- Parents frequency in the unit. - Participation.	Family-centered care program: nursing attitude change. Increase parents participation.	Results not generalizable. Subjective experiences.
Coasts et al. (2018) USA	Qualitative	Describe nursing perceptions about benefits and challenges of providing family-centered care in the NICU.	*n* = 10 NICU nurses.	- NICU environment. - Provision of critical care. - Nurse and family stressors. - Communication challenges and strategies. - Family participation in care and decision making.	Nurses find family-centered care beneficial. But the changes created in the NICU posed a challenge in the provision of care. Policy changes must be made including nurses.	They have no limitations.
Toivonen et al. (2019) Finland	Qualitative	Explore professional perception regarding the implementation of the parent training program in neonatal care.	*n* = 19 NICU managers. *n* = 32 nurses.	- NICU Features: N° beds N° Patients/year Gestation weeks N° nurses N° neonatologists - Professional Features: Gender Work experience Middle Ages	Nurses commitment and motivation to change their role, key in program implementation, parents as partners in the care of the Newborn (NB).	Unable to include NICU physicians. Subjective experiences of nurses and managers. No examination of parental experiences.
Gilstrap et al. (2020) USA	Qualitative	The significance of a new organization in the NICU for nursing.	*n* = 14 nurses	Educating parents: informing to improve the health and well-being of premature infants, promoting care participation Promote open communication: simple language. Constant contact	Nurses rely on communication to build knowledge of parents. Nurses empower FCC. Managers encourage: organizational structures, more training resources.	Only female nurses participated. The study was conducted in only one hospital. There is no paternal perception.
Heidari et al. (2020) Iran	Qualitative (six months duration)	Understand the perception of nurses about FCC in the NICU.	*n* = 18 nurses *n* = 2 NICUS N-III.	Stay 24 h Only mothers (not parents or grandparents) Training spaces Nurse Training	Poor hospital facilities. Little staff to form FCC. Greater workload, parent dissatisfaction.	The participants were women nurses. It would be interesting to have more nurses participate.
Mirlashari et al. (2020) Iran	Qualitative Thematic content analysis approach	Nursing perception understanding of implementing FCC in the NICU.	*n* = 40 professionals *n* = 25 nurses *n* = 15 neonatologists	Research nurses ‘and physicians’ perspectives on implementing FCC in the NICU: Imbalance of power Psychosocial problems Structural limitation	FCC implementation on NICU is determined: -Cultural, legal and operational challenges. Nurses and doctors are positioned as leaders and facilitators of FCC implementation in the NICUs.	Health policy and operational changes are required to implement FCC in NICU.

## List of Abbreviations

NICU	neonatal intensive care unit
PICU	pediatric intensive care unit
NB	newborn
FCC	family-centered care
NIDCAP	newborn individualized developmental care and assessment program
WHO	World Health Organization
HQ	high quality
MQ	medium quality
LQ	low quality
